# Anatomo-physiological basis and applied techniques of electrical neuromodulation in chronic pain

**DOI:** 10.1186/s44158-024-00167-1

**Published:** 2024-05-02

**Authors:** Giusy Guzzi, Attilio Della Torre, Andrea Bruni, Angelo Lavano, Vincenzo Bosco, Eugenio Garofalo, Domenico La Torre, Federico Longhini

**Affiliations:** 1 Neurosurgery Department, “R. Dulbecco” Hospital, Department of Medical and Surgical Sciences, “Magna Graecia” University of Catanzaro, Catanzaro, Italy; 2 Anesthesia and Intensive Care Unit, “R. Dulbecco” Univesity Hospital, Department of Medical and Surgical Sciences, Magna Graecia University, Viale Europa, Catanzaro, 88100 Italy

**Keywords:** Chronic pain, Pain mechanisms, Neuromodulation, Deep Brain Stimulation, Direct Cortical Stimulation, Spinal Cord Stimulation, Motor Cortex Stimulation, Peripheral Nerve Stimulation, Transcranial Focused Ultrasound

## Abstract

Chronic pain, a complex and debilitating condition, poses a significant challenge to both patients and healthcare providers worldwide. Conventional pharmacological interventions often prove inadequate in delivering satisfactory relief while carrying the risks of addiction and adverse reactions. In recent years, electric neuromodulation emerged as a promising alternative in chronic pain management. This method entails the precise administration of electrical stimulation to specific nerves or regions within the central nervous system to regulate pain signals. Through mechanisms that include the alteration of neural activity and the release of endogenous pain-relieving substances, electric neuromodulation can effectively alleviate pain and improve patients' quality of life. Several modalities of electric neuromodulation, with a different grade of invasiveness, provide tailored strategies to tackle various forms and origins of chronic pain. Through an exploration of the anatomical and physiological pathways of chronic pain, encompassing neurotransmitter involvement, this narrative review offers insights into electrical therapies’ mechanisms of action, clinical utility, and future perspectives in chronic pain management.

## Introduction

The International Association for the Study of Pain (IASP) defines pain as "an unpleasant sensory and emotional experience associated with, or resembling that associated with, actual or potential tissue damage" [1]. In 2013, the IASP established a task force to develop and update a classification of pain disorders. This effort led to the inclusion of a chronic pain classification in the 2019 edition of the International Classification of Diseases (ICD-11), formally adopted by the World Health Organization [2, 3]. Chronic pain is defined by a duration of at least three months or beyond the usual healing period. It can be further categorized into nociceptive, neuropathic, and nociplastic pain types [4]. Chronic pain presents a significant public health challenge, affecting millions of patients and incurring substantial medical expenses and lost productivity [5, 6]. According to the Global Burden of Disease Study, tension-type headache, migraine, low back pain, neck pain, diabetic neuropathy are among the most common and prevalent chronic pain syndromes in the population, increasing their years lived with disability [6].

In a certain percentage of patients, conventional treatments, including surgery, pharmacological therapies, combined with psychological, physical and occupational therapies, fail [7–9]. In these patients, neuromodulation may have a role in treatment. Neuromodulation refers to a broad category of techniques or therapies that modulate the activity of the nervous system to achieve therapeutic effects through chemical (i.e. pharmacological) interventions or electrical stimulation (i.e. neuromodulation). Neurostimulation, which involve modifying or stimulating nerve activity through targeted electrical at specific neurological sites, is increasingly utilized in patients with chronic pain of varied origins [10]. These modalities represent the evolving landscape for chronic pain management, each offering unique advantages and considerations [11].

This review aims to provide a comprehensive understanding of the neuroanatomy and neurophysiology of chronic pain, with an emphasis on the role of electrical neuromodulation in its treatment.

### Neuroanatomy and neurophysiology of chronic pain

Pain pathways are intricate and dynamic systems comprising sensory, cognitive, and behavioral components. These mechanisms have evolved to recognize, integrate, and coordinate protective responses to noxious stimuli. They encompass both primitive spinal reactions and the nuanced emotional experiences consciously identified as pain in humans [12].

**Fig. 1:**
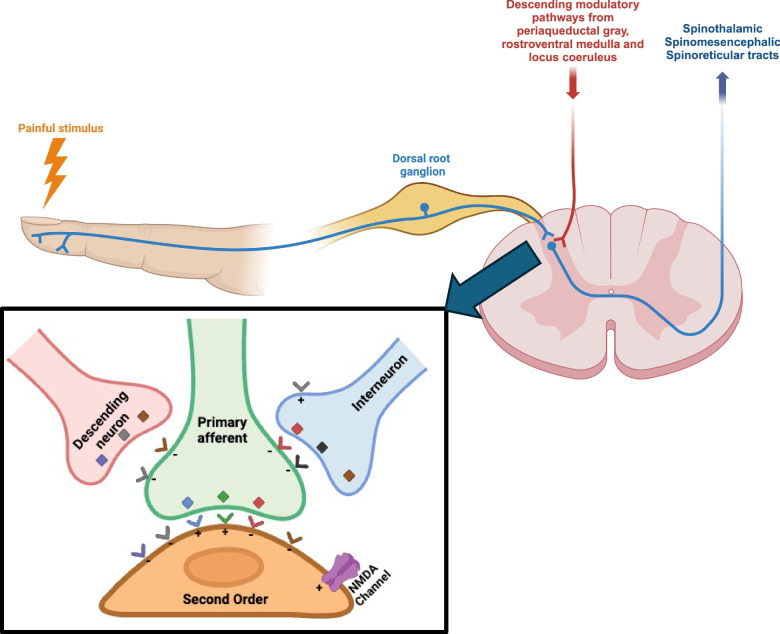
Anatomical pathways of chronic pain and neurotransmitters. The figure depicts the ascending and descending anatomical pathways of pain modulation. In the inner panel, neurotransmitters (depicted as squares) and receptors (depicted as V shapes) are illustrated. These include GABA (red), glycine (black), opiates (brown), norepinephrine (gray), glutamate (green), substance P (light blue), and serotonin (violet), each with their respective inhibitory (-) or excitatory ( +) activity indicated

In addition to the peripheral pain pathways, which include receptors and neural fibers, the central nervous system (CNS) pain pathways involve a network of structures. These structures comprise the spinal cord, thalamus, amygdala, hypothalamus, periaqueductal grey (PAG) matter, basal ganglia, insular and cingulate cortices, as well as the sensory and motor cortices [13]. Figure 1 illustrates a schematic representation of pain pathways.

#### Pain signaling from periphery to ascending pathways

The intricate process of a painful stimulus begins in the periphery with specialized nociceptors activated by stimuli like pressure, extreme temperatures, or tissue damage. Nociceptors transmit the pain stimulus to primary afferent fibers, responsible for carrying sensory information from the periphery to the CNS. Classified based on diameter and conduction velocity, two basic types of nerve fibers exist: myelinated Type A and unmyelinated Type C. Pain transmission occurs through two types: rapid (associated with Aδ-fibers) and slow (related to polymodal receptors and C fibers), each transmitting pain at different speeds. The cell bodies of sensory neurons are situated in the dorsal root ganglia (DRG); these neurons are capable of encoding and transmitting information derived from external stimuli. As these primary afferent fibers approach the spinal cord, they enter through the dorsal roots (i.e., dorsal root entry zone) [12, 13]. Inside the spinal cord, unmyelinated and small myelinated axons project laterally to enter the Lissauer tract, a marginal zone or layer of the dorsal horn. Here, they synapse with neurons in the dorsal horn. Aδ fibers ascend three to four segments in the Lissauer tract before terminating in the Rexed’s lamina I, II outer, or V. In contrast, C fibers typically ascend one segment before ending in lamina II.

One of the principal conduits in this relay of pain signals is the spinothalamic tract. This major ascending pathway serves as a dedicated highway for transmitting pain signals to the brain. The second-order neurons of the spinothalamic tract, crucial intermediaries in this relay, undergo a remarkable anatomical phenomenon known as decussation. These neurons cross over to the contralateral side of the spinal cord before ascending towards the thalamus, a central hub for sensory processing [14]. The thalamus acts as a sensory relay station, receives the pain signals, and orchestrates their distribution to various cortical areas. From the thalamus, projections fan out to regions of the brain responsible for the conscious perception of pain. These cortical areas, intricately connected and functioning in concert, give rise to the multifaceted experience of pain that encompasses its sensory, emotional, and cognitive dimensions [14].

The spinoreticular tract, diverging from traditional sensory pathways, projects to the reticular formation in the brainstem, influencing emotional and autonomic aspects of pain perception. Additionally, the spinocerebellar tract, known for proprioceptive transmission, also contributes to nociception and motor responses to pain, further intertwining sensory perception and motor output [13].

#### Pain modulation from descending pathways

As pain signals ascend from the periphery to the brain through the intricate pathways, a parallel network of descending modulatory pathways operates in concert, finely tuning the transmission and perception of pain, maintaining a delicate balance between the intensity of noxious stimuli and the perceptual experience of pain.

One of the most prominent players in the descending modulatory orchestra is the descending inhibitory pathway. This pathway exerts its influence through a series of neural structures, i.e. the PAG, situated around the cerebral aqueduct in the midbrain, and the rostroventral medulla (RVM), a region placed in the brainstem. These regions control the release of endogenous opioids and other neurotransmitters that act as pain inhibitors [15, 16]. In particular, the PAG integrates information from various brain regions; its activation largely reduces perception of pain [17].

Descending from the PAG, the inhibitory signals make their way to the RVM. The RVM acts as a relay station, forwarding the inhibitory commands to the spinal cord. Within the spinal cord, these commands target nociceptive neurons, the primary relay stations for pain signals. The release of endogenous opioids, such as enkephalins and endorphins, is a hallmark of this inhibitory process [18]. These opioids bind to receptors on nociceptive neurons, effectively dampening their activity and reducing the transmission of pain signals. Beyond endogenous opioids, neurotransmitters like serotonin and norepinephrine also play crucial roles in the descending inhibitory pathway. The release of these neurotransmitters contributes to the overall suppression of nociceptive signals. Serotonin is known for its involvement in pain modulation and mood regulation, highlighting the intricate interplay between sensory and emotional components in pain perception[18].

The descending inhibitory pathway not only reduces the transmission of pain signals but also contributes to the phenomenon of "pain gating". The Gate Control Theory, proposed by Melzack and Wall, suggests that non-nociceptive inputs can modulate pain perception [19].

The dorsal column of the spinal cord comprises various neural fibers, with certain types such as Aδ and C fibers being triggered by painful stimuli, while the larger Aβ fibers serve to suppress the transmission of these signals. According to the "Gate Control Theory," the interplay between the activity of Aδ and C fibers versus that of Aβ fibers dictates the perception of painful stimuli [7].

RVM is also known for the presence of "on-cells," which release neurotransmitters such as Substance P, glutamate, and norepinephrine. These neurotransmitters have the effect of amplifying the activity of nociceptive neurons in the spinal cord rather than inhibiting them [18].

#### Neurotransmitters of pain

Multiple neurotransmitters participate in both transmitting and modulating pain signals, exerting effects that can either enhance or suppress the perception of pain [20].

Substance P, a neuropeptide functioning as an excitatory neurotransmitter, is primarily synthesized and secreted by nociceptive neurons, transmitting and modulating pain signals throughout the CNS [21]. In the spinal cord, Substance P is released both at synaptic junctions and non-synaptic sites within the dorsal horn. It binds to tachykinins (NK1, NK2 and NK3) receptors located on lamina 1 neurons and the dendrites of lamina 5 neurons [22]. This interaction facilitates the transmission of pain signals from peripheral nerves to the CNS and assists in relaying these signals to higher brain regions responsible for processing pain perception. Additionally, Substance P can induce the release of other neurotransmitters, such as glutamate, thereby intensifying the pain signals [23]. Moreover, Substance P contributes to the emotional and affective dimensions of pain perception, influencing brain areas associated with mood regulation, stress responses, and emotional processing [24].

Glutamate is another excitatory neurotransmitter in the CNS involved in the conduction of pain signals by activating amino-3-hydroxy-5-methyl-4-isoxazole-propionic acid (AMPA) and N-Methyl-D-Aspartate (NMDA) receptors on postsynaptic neurons in the dorsal horn of the spinal cord [25]. This activation induces the depolarization of postsynaptic neurons, generating action potentials and transmitting pain signals along ascending pathways to the brain centers [26]. NMDA receptors are crucial for amplifying and modulating pain signals, especially in chronic pain conditions. Unlike AMPA receptors, NMDA receptors necessitate both glutamate binding and postsynaptic depolarization for full activation of their ion channels. This property allows NMDA receptors to integrate synaptic activity and contribute to the phenomenon of central sensitization, wherein pain signals are amplified and prolonged in chronic pain conditions [27].

Calcitonin Gene-Related Peptide (CGRP) is a neuropeptide synthetized and released from sensory nerves in response to noxious stimuli, inflammation, and tissue injury. CGRP is a potent vasodilator, and it contributes to inflammation and hypersensitivity [28]. In addition, it enhances the excitability of sensory neurons and promotes the release of other neurotransmitters, such as substance P [29]. CGRP has received significant attention in the context of migraine since elevated levels have been found in the blood and cerebrospinal fluid during attacks. Therefore, CGRP has become a target for novel treatments for these patients [30].

Norepinephrine is released from descending pathways (particularly from the locus coeruleus), having both inhibitory and excitatory effects on pain transmission. Its net effect depends on the receptors it acts upon, with α2-adrenergic receptors generally inhibiting pain signals [31, 32]. Direct stimulation of the PAG or RVM elevates norepinephrine levels in the cerebrospinal fluid, then modulating pain transmission in the spinal cord by inhibition of the release of glutamate and substance P [33].

Serotonin, also known as 5-hydroxytryptamine (5-HT), is another neurotransmitter involved in pain modulation. It exhibits both excitatory and inhibitory effects on pain transmission, which are contingent upon the receptor subtype activated and the neural pathways engaged [34]. 5-HT exerts its effects through multiple receptor subtypes (including 5-HT1, 5-HT2, 5-HT3, and so on), differently distributed throughout the nervous system and with distinct functional properties. Of note, 5-HT is characterized by an excitatory or inhibitory effect on pain transmission according to the activated subtype of receptors [35]. For example, 5-HT2 and 5-HT3 receptors enhance pain transmission by increasing neuronal excitability and amplifying the signaling of pain pathways. Conversely, 5-HT1 receptors suppress the release of excitatory neurotransmitters, dampen neuronal activity, and reduce the transmission of pain signals, resulting in pain relief [34]. It's worth noting that in the typical condition, 5-HT concentrations are relatively low, with a certain level of facilitation mediated by 5-HT3 and 5-HT2 receptors. Subsequent elevations in spinal 5-HT then result in the inhibitory actions of the 5-HT7 receptor [35].

5-HT exerts regulatory effects on pain pathways across various levels of the nervous system. Apart from its role in the spinal cord, it also impacts pain processing in several brain regions associated with pain and anxiety, including the thalamus, frontal cortex, amygdala, and brainstem [36].

Gamma-Aminobutyric Acid (GABA) is the principal inhibitory neurotransmitter in the CNS. GABA produces its inhibitory effects by inducing hyperpolarization of postsynaptic neurons, thereby increasing the negativity of the neuron's membrane potential. This hyperpolarization reduces the likelihood of the neuron generating an action potential, leading to decreased excitability and limiting the propagation of signals along the neural pathway [37]. Within pain modulation, GABAergic activity mitigates pain signals, regulating both the intensity and duration of pain perception [24]. In the spinal cord, GABA is released onto postsynaptic neurons subsequent to receiving inputs from primary nociceptive neurons, exerting inhibitory control [38]. Conversely, GABA modulates various regions in the brain such as the thalamus, where it inhibits transmission to the cortex, as well as the somatosensory cortex, insula, and anterior cingulate cortex. Furthermore, GABA influences descending pain pathways including the PAG and RVM [39]. Additionally, GABAergic inhibition extends to the emotional and affective dimensions of pain processing, impacting brain regions like the amygdala and prefrontal cortex, thus modulating emotions such as fear, anxiety, and depression [24].

Endorphins and enkephalins, endogenous opioids synthesized in diverse CNS locales such as the hypothalamus, pituitary gland, and spinal cord, serve as neurotransmitters and neuromodulators, predominantly triggered by stress, pain, and physical exertion [40]. A key function of these compounds is pain signal inhibition, facilitated by their binding to mu (μ), delta (δ), and kappa (κ) opioid receptors on neurons in the spinal cord and brain, initiating intracellular cascades that lead to neuronal suppression [41]. Within the spinal cord, endorphins and enkephalins hinder the release of substance P and glutamate while diminishing the excitability of pain-transmitting neurons, thereby modulating the transmission of pain signals from the periphery to the brain. Moreover, these opioids exert their analgesic effects across various brain regions, including the PAG, RVM, thalamus, and limbic system [42].

#### Peripheral and central sensitization

Peripheral sensitization is a condition defined by the IASP as a state of “Increased responsiveness and diminished threshold of nociceptive neurons in the periphery to the stimulation of their receptive fields” [43, 44]. This phenomenon occurs because of chemical mediators released by nociceptors and various non-neuronal cells, such as mast cells, basophils, platelets, macrophages, neutrophils, endothelial cells, keratinocytes, and fibroblasts, at the site of tissue injury or inflammation. A plethora of signaling molecules is involved in this process, including protons, ATP, prostaglandins (PGE2), thromboxanes, leukotrienes, endocannabinoids, growth factors (neurotrophins, granulocyte- or granulocyte-macrophage colony-stimulating factors), cytokines (IL6, IL1β, TNFα), chemokines, neuropeptides (CGRP, substance P, bradykinin, histamine), lipids, and various proteases [45–49].

Conversely, central sensitization is a complex phenomenon defined by an amplification of neural signaling within the CNS that elicits pain hypersensitivity [50]. In particular, it is marked by lasting changes in the excitability of second-order neurons within the spinal cord, induced by increased afferent activity. This results in significant alterations to the somatosensory system itself [51]. The profound significance of this concept was emphasized by the description of the long-term potentiation in the hippocampus, revealing that synchronous high-frequency input enhances synaptic efficacy [52]. Central sensitization has been postulated to contribute in several chronic pain syndromes, such as rheumatoid arthritis, osteoarthritis, temporomandibular disorders, fibromyalgia, musculoskeletal disorders, headache, neuropathic pain, complex regional pain syndrome [50, 53].

### Neurostimulation techniques

The fundamental principle of neurostimulation in chronic pain revolves around precisely delivering electrical impulses to modulate nervous system activity, thereby modifying pain perception, and providing relief. This approach aims to interrupt or dampen pain signals along neural pathways, by either stimulating specific nerves to block pain transmission or influencing the brain's processing of pain signals [54]. More precisely, in the complexity of anatomical and physiological mechanisms of chronic pain, neurostimulation, in its various forms, interferes with the hyperexcitability of circuits involved in pain processing (such as the spinal cord stimulation and peripheral nerve stimulation) [55, 56], enhances endogenous pain inhibition (such as the motor cortex stimulation and the transcranial magnetic stimulation) [57–59], or modulates the affective component of pain [54, 60, 61]. Neurostimulation is an alternative or adjunctive approach to manage chronic pain, particularly when medical or surgical treatments have been ineffective or associated with undesirable side effects [54].

**Fig. 2:**
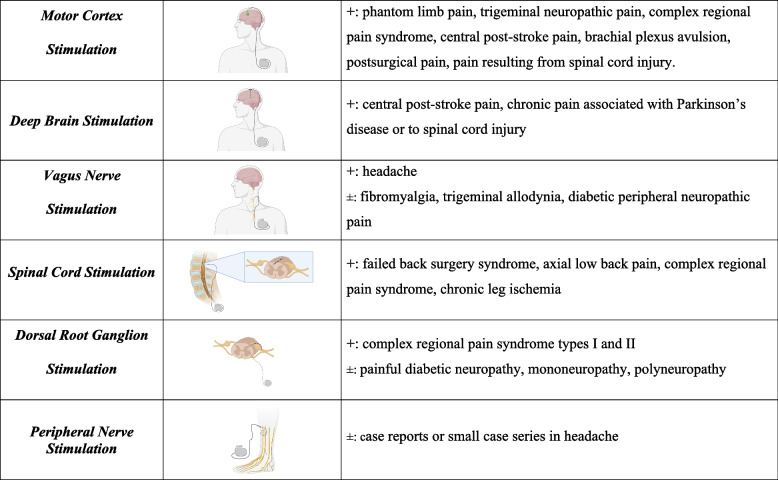
Invasive neurostimulation techniques. The invasive techniques for neurostimulation of chronic pain are depicted alongside their current indications with clear ( +) or unclear ( ±) evidence. The specific characteristics of each modality are explained in the text

**Fig. 3:**
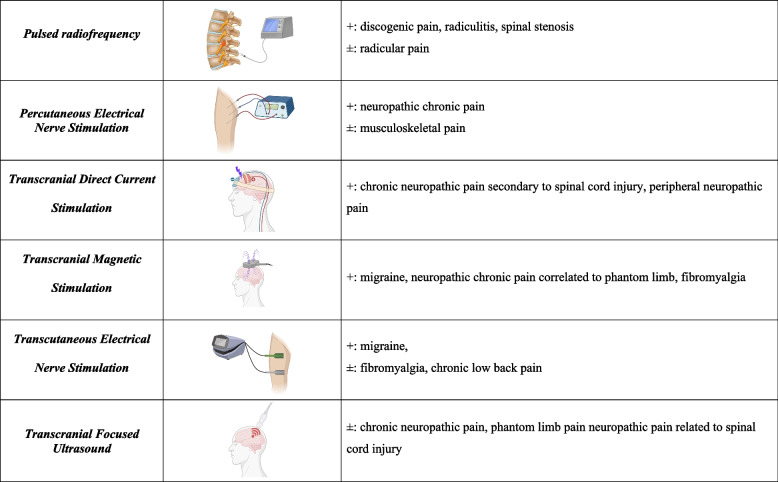
Mini-invasive and non-invasive neurostimulation techniques. The mini-invasive (i.e. pulsed radiofrequency therapy and percutaneous electrical nerve stimulation) and non-invasive (i.e. transcranial direct current stimulation, transcranial magnetic stimulation, transcutaneous electrical nerve stimulation and transcranial focused ultrasound) techniques for neurostimulation of chronic pain are schematized in the representation alongside their current indications with clear ( +) or unclear ( ±) evidence. The specific characteristics of each modality are explained in the text

Neurostimulation therapies can be classified by invasiveness. Invasive methods involve surgical implantation of electrodes or devices, including motor cortex stimulation, deep brain stimulation, vagus nerve stimulation, spinal cord stimulation, and peripheral nerve stimulation (Figure 2). Minimally invasive modalities, like pulsed radiofrequency therapy and percutaneous electrical nerve stimulation are less intrusive but involve some intervention (Figure 3). Lastly, noninvasive techniques, such as transcranial direct current stimulation, transcranial magnetic stimulation, transcutaneous electrical nerve stimulation, transcranial focused ultrasound, do not require any surgical intervention or implantation and are typically administered externally to the body (Figure 3) [54].

#### Motor cortex stimulation

Motor Cortex Stimulation (MCS) consists in the implantation of an array of electrodes directly onto the motor cortex of the brain contralateral to the painful side [62, 63]. Particularly, after craniotomy, the electrode array is positioned in the precentral gyrus or central sulcus corresponding to the painful area either in the epidural or subdural spaces [64, 65]. Although there is not an evidence-based difference, opting for subdural lead placement may be more reasonable due to the reduced distance between the leads and the brain cortex compared to epidural placement [66].

MCS is theorized to alleviate pain by influencing both the emotional dimension of pain and inhibiting pain signals at different neural levels. Specifically, MCS relieves pain by activating the perigenual cingulate and orbitofrontal cortex, thus modulating the emotional component of pain. Simultaneously, MCS inhibits pain impulses at the spinal cord level by activating the PAG [67].

The research of MCS as a potential treatment for various neuropathic chronic pain populations has yielded inconsistent results. This variability is primarily attributable to small sample sizes and study designs of lower quality, suggesting that MCS is efficient only in specific subgroups of patients. Noteworthy, MCS has demonstrated effectiveness in addressing chronic pain associated with phantom limb pain, trigeminal neuropathic pain, complex regional pain syndrome, central post-stroke pain, brachial plexus avulsion, postsurgical pain, and pain resulting from spinal cord injury [66, 68–74].

#### Deep brain stimulation

Deep Brain Stimulation (DBS) consists in the surgical implantation of thin electrodes within specific area of the brain to be stimulated. In case of chronic pain, electrodes are generally positioned in the PAG, in the periventricular gray matter, the ventralposterior medial or the ventral posterior lateral nuclei of the thalamus (contralateral to the side of pain), the anterior cingulate cortex or the posterior insula [75, 76]. The positioning generally required a frame-based or frame-less stereotactic technique, targeting the area with a magnetic resonance imaging on the day before surgery and, in some centers, with intraoperative microelectrode recordings. Electrodes are then connected to an impulse generator that neuromodulate the target area [77].

When PAG or thalamus are stimulated, DBS seems to promote the secretions of endogenous opioids (i.e. beta-endorphin and methionine enkephalin) [78, 79]. Beside these reports, the other underlying mechanisms are still obscure.

Clinical studies have demonstrated a significant improvement in patients with central post-stroke pain [80, 81], in chronic pain associated with Parkinson’s disease [82, 83] and in those patients with spinal cord injury [84]. The most favorable outcomes in DBS, characterized by effects surpassing 50%, were observed when stimulating the somatosensory thalamus in individuals experiencing peripheral neuropathic pain [85, 86]. Nevertheless, the current trend indicates a decline in the number of patients undergoing implantation, primarily attributed to the emergence of new and less invasive treatment modalities. In addition, although considered safe, DBS is affected by adverse event in 8–9% of patients, including lead fractures, wound infections, intra-operative seizure and postoperative burr hole site erosion [87]. It is noteworthy that individuals exhibiting severe, debilitating neuropathic pain accompanied by verifiable pathology, resistant to conventional treatments, appear to be the most suitable candidates for this approach.

#### Vagus nerve stimulation

Vagus Nerve Stimulation (VNS) is a therapeutic method that employs electrical impulses to activate the vagus nerve, a component of the autonomic nervous system. Although widely recognized for its efficacy in treating epilepsy [88] and depression [89], VNS has also been investigated in chronic pain [90]. VNS involves the surgical placement of a stimulator device, delivering regular electrical impulses, connected to electrodes stimulating the vagus nerve. More recently, transcutaneous VNS and minimally invasive form of percutaneous VNS have been also developed [90].

The vagus nerve’s afferent pathway initiates from vagal afferents that supply the cervical, thoracic, and abdominal organs. Primarily traversing the nucleus of the solitary tract, it ultimately reaches higher brain regions, including the thalamus, hypothalamus, parabrachial nucleus, PAG, amygdala, and locus coeruleus [91–94]. The stimulation of vagus nerve modulates the descending serotoninergic and noradrenergic neurons, modulating the central sensitization and reducing the pain transmission [90]. VNS also induces anti-inflammatory effects through diverse pathways, such as the cholinergic anti-inflammatory pathway, the hypothalamic–pituitary–adrenal axis pathway, and the production of specialized pro-resolving mediators. Specifically, VNS initiates the release of acetylcholine from Vagus Nerve efferents in the coeliac ganglia and norepinephrine from the splenic nerve, subsequently promoting further release of acetylcholine [95]. Following its release, acetylcholine binds to the α7 nicotinic Ach receptors present on macrophages, leading to a decrease in cytokine production [95, 96]. Additionally, VNS activates the hypothalamic–pituitary–adrenal axis, triggering the release of adrenocorticotropic hormone. This hormone, in turn, acts on the adrenal glands, promoting the synthesis of cortisol [97]. Furthermore, VNS has the potential to induce the production of specialized pro-resolving mediators [98], which act on receptors expressed on immune cells, gliacytes, and neurons, thereby exerting a modulatory effect on inflammation and neuroinflammation [99–101].

Therapeutic effects on chronic pain have been casually discovered during the application of VNS for epilepsy. Some epileptic patients reported a reduction in headache frequency and intensity that after VNS implantation [102–104]. Following these first reports, several studies demonstrated the efficiency of VNS in preventing and reducing the intensity of headache, either with invasive implantation [105] and during transcutaneous stimulation [106–109]. Given the efficacy in modulation of central sensitization [90] and in depressive disease [89], VNS was reported to successfully reduce symptoms in patients with fibromyalgia [110], although these results were not further confirmed by other investigations [111]. VNS has been also investigated in other scenarios of chronic neuropathic pain (i.e. trigeminal allodynia and diabetic peripheral neuropathic pain) in animal models [112–115]. However, data in patients still lack and clinical studies are needed to validate the positive findings reported by animal studies.

#### Spinal cord stimulation

Spinal Cord Stimulation (SCS) is a neuromostimulation technique designed to address and alleviate neuropathic chronic pain. This method involves an implanted generator that produces pulsed electrical signals, which are then delivered to a specific area of the spinal cord through electrodes implanted within the epidural space [7]. Advancements in SCS techniques and equipment are ongoing, with dedicated literature available elsewhere [7]. SCS operates through neurophysiological and neurochemical mechanisms rooted in the "gate control theory” for pain transmission [7, 19].

SCS disrupts the processing of nociceptive signals through the lateral spinothalamic tract, influencing supraspinal brain centers like the ventral posterior nucleus of the thalamus, somatosensory cortex, cingulate cortex, and insula [116, 117]. Orthodromically, SCS depolarizes Aβ fibers cranially, controlling supraspinal centers such as the cuneate and gracile nuclei [118, 119]. After supraspinal integration, descending feedback loops from the locus coeruleus [120], nucleus raphe magnus [121], and rostral ventromedial medulla [122] modulate and control the spinal nociceptive signal at the "spinal gate" via serotonergic and noradrenergic projections to the dorsal horn [118, 119].

In terms of neurochemical mechanisms, SCS modulates gamma-aminobutyric acid (GABA) [123], serotonin [124], acetylcholine and norepinephrine [125, 126]. Studies in animal models show increased intraspinal release of GABA [127, 128] and attenuation of glutamate and aspartate responses [129]. Notably, GABA type "b" receptors play a crucial role, suggesting the potential for intrathecal baclofen administration to enhance SCS analgesia [130]. SCS also elevates serotonin and substance P release [124], enhances dynorphin and enkephalin expression [131], and decreases neuronal excitability and spinal pain transmission by activating 5-HT2A, 5-HT3, and 5-HT4 receptors [132]. Additionally, SCS promotes analgesia by modulating cholinergic and adrenergic neurotransmission, releasing acetylcholine and noradrenaline in the dorsal horn of the spinal cord [125, 126].

SCS is considered for those patients experiencing chronic pain, particularly when other conventional treatments (including pharmacological therapies, surgical treatments or physiotherapy) have proven ineffective or are associated with intolerable side effects. To define the indication of SCS implantation, the selection of the patient is fundamental for its success [133]. Firstly, SCS should be contemplated within two years of symptom onset, following the ineffectiveness of all standard therapies [134, 135]. It should be mentioned that the time between initial pain diagnosis and SCS implantation is still debated. In a study by Kumar et al., a time to SCS treatment < 2 years was characterized by a higher long term success rate (around 85%), declining precipitously at the lengthening of the diagnosis to implantation interval [136]. Another study by the same group reported that the greatest improvement in pain relief was obtained in case of SCS implantation within one year from the onset of symptoms [137]. However, several studies have shown a symptom-to-implantation waiting time from 5 to 6 years [138, 139]. Secondly, individuals with underlying psychiatric conditions, complete cognitive impairment, psychological comorbidities, or substance abuse are ineligible for SCS implantation [140]. However, if there is partial cognitive impairment, SCS may be considered, with a preference for non-rechargeable over rechargeable implantable pulse generators [134]. Thirdly, SCS is a viable option for neuropathic pain conditions (e.g., failed back surgery syndrome, arachnoiditis, complex regional pain syndrome, causalgia, peripheral neuropathy, chronic radiculopathy), while its efficacy is limited for nociceptive symptoms or central neuropathic pain [134]. SCS is strongly recommended in specific scenarios, including failed back surgery syndrome without neurologic progression [141], axial low back pain [142] and complex regional pain syndrome [143]. Furthermore, SCS is recommended for chronic refractory angina unresponsive to maximal medical therapy, bypass surgery, and percutaneous angioplasty of the legs [144], as well as for peripheral artery disease or non-reconstructable critical leg ischemia [145]. In particular, SCS is employed in patients experiencing chronic leg ischemia that is not suitable for open surgical or endovascular intervention [146]. This condition is typically characterized by either rest pain alone (Fontaine stage III) or the presence of rest pain accompanied by arterial ulcers less than 3 cm in diameter (Fontaine stage IV) [147].

#### Dorsal root ganglion stimulation

Dorsal root ganglion (DRG) stimulation resembles SCS, but with devices specifically design to target the first-order sensory neurons located in the DRG. The DRG's advantageous position within the epidural space, immersed in cerebral spinal fluid, makes it an ideal target for stimulation, allowing precise targeting of chronic pain. DRG stimulation applies an electrical field directly over the nuclei of primary afferent neurons, enabling modulation prior to signal propagation within the spinal cord. This modulation potentially includes inhibition at synapses with second-order neurons in the dorsal horn [148].

Recent pre-clinical and clinical research is shedding light on the mechanisms and therapeutic effects of DRG stimulation, highlighting differences compared to SCS. Unlike SCS, DRG stimulation does not elevate the inhibitory GABA neurotransmitter in either the dorsal horn or the DRG itself [149, 150]. The most accepted mechanism of DRG stimulation involves impeding the transmission of afferent pain signals and ectopic action potential transmission, supported by basic science research [151–153]. However, its effects on multi-dermatomal conditions with a single lead placement may depend on orthodromic propagation, a mechanism often discussed in SCS but less so in DRG stimulation [154, 155]. DRG stimulation is believed to activate Aδ-, Aβ-, and C-fiber low threshold mechanoreceptor fibers, utilizing the endogenous opioid system to modulate touch and pain processes at clinically utilized frequencies [156–158]. Furthermore, DRG stimulation has effects on the sympathetic nervous system, including antidromic effects in the treatment of peripheral vascular disease, reduction in blood pressure, and attenuation of neuroinflammation [159–162].

In 2019, the Neuromodulation Appropriateness Consensus Committee highlighted the significant efficacy of DRG stimulation in treating both complex regional pain syndrome types I and II, as well as focal neuropathic pain with identifiable pathology [163]. In fact, a large prospective randomized controlled trial, comparing SCS to DRG stimulation in patients affected by complex regional pain syndrome types I and II, demonstrated that 81.2% of DRG patients achieved ≥ 50% pain relief compared to 55.7% in the SCS arm [164].

Today the DRG stimulation is mainly accepted for the treatment of complex regional pain syndrome types I and II, although it has been attempted in several other forms of neuropathic chronic pain (including painful diabetic neuropathy, mononeuropathy, polyneuropathy) with for these latter a low-quality study and limited evidence [148, 165].

#### Peripheral nerve stimulation

Peripheral Nerve Stimulation (PNS) consists in the delivery of electrical impulses through electrodes surgically implanted near peripheral nerves, to modulate the transmission of pain signals along these nerves [166]. As per the SCS, PNS engages neurophysiological and neurochemical mechanisms based on the "gate control theory" of pain transmission, as above discussed [7, 19].

PNS can be implanted in various anatomical regions, including the upper extremities (brachial plexus, suprascapular nerve, axillary nerve, radial nerve, median nerve, or ulnar nerve), lower extremities (sciatic nerve, obturator nerve, femoral nerve, lateral femoral cutaneous nerve, genicular nerve, saphenous nerve, common peroneal nerve, tibial nerve, sural nerve, and superficial peroneal nerve), as well as other nerves in the head (occipital nerve, trigeminal nerve, and supraorbital nerve), trunk, abdomen, back, or pelvis (medial branch nerve, ilioinguinal nerve, iliohypogastric nerve, genitofemoral nerve, cluneal nerve, and pudendal nerve) [166].

The available evidence on PNS in the head and neck region has predominantly centered in case reports or small case series around the stimulation of occipital nerves for the treatment of headache disorders with a positive therapeutic effect [167–170]. On the opposite, the evidence for facial pain is still weak, lacking high quality studies [171–174]. Indeed, the current guidelines do not recommend PNS for chronic facial pain, whereas they state that PNS can be considered in case of chronic migraine headache after failure of conventional treatments [175].

According to the guidelines [175], PNS can also be considered as a treatment option for mononeuropathies affecting the upper extremity, failed back surgery syndrome, and chronic low back pain (supported by substantial evidence). Additionally, it may be explored in cases of radiculopathy and post-herpetic neuralgia, although the evidence for these conditions is limited. Furthermore, PNS shows promise in managing neuropathic pain in the lower extremities, including post-amputation pain, as well as in individuals with Complex Regional Pain Syndrome Type I/II or peripheral causalgia (while acknowledging that SCS remains the preferred neurostimulation option) [175].

#### Pulsed radiofrequency therapy

Pulsed Radiofrequency Therapy (PRF) is a medical intervention employed in the management of chronic pain conditions. In contrast to continuous radiofrequency, which subjects target nerves or tissues to sustained high temperatures (70–90 °C) through electrical stimulation [176], PRF utilizes electrical pulses delivered to specific nerves via an electrode generating localized thermal effects without causing substantial tissue damage [177]. Specifically, PRF employs a radiofrequency current characterized by alternately repeated electrical stimulation with a short duration (around 20 ms) followed by a resting phase (around 480 ms). This alternating pattern induces a temperature on the target tissue that remains below 42 °C [178].

PRF induces a targeted and prolonged reduction in spinal sensitization mediated by C-fibers. As a result, it effectively inhibits the transmission of pain signals from peripheral nerves to the central nervous system [179, 180]. PRF is reversible and temporary, it provides relief for a variable duration, and the procedure may be repeated if necessary [178].

PRF is commonly applied to peripheral nerves, DRG, or other nerve structures contributing to chronic pain. Therefore, PRF is often used to modulate neural activity and disrupt pain signals without causing tissue damage, whereas continuous radiofrequency creates thermal lesions that disrupt nerve function and alleviate pain [176–178]. Based on these different effects, PRF is commonly employed for conditions where nerve-related pain is the primary concern, such as neuropathic pain syndromes or neuralgias. On the opposite, continuous radiofrequency is frequently used for conditions characterized by localized pain arising from specific anatomical structures, such as facet joints or sacroiliac joints [176–178].

The most important efficacy of PRF has been shown in the treatment of postherpetic neuralgia [181] targeting the areas near the DRG via the angulus costae [182] or paravertebral puncture [183] or targeting the intercostal nerves [184, 185].

In recent findings, the literature has indicated limited and weak evidence for the use of PRF on the lumbar DRG for radicular pain [186]; in facets’ pain, PRF has been also considered to be inferior to continuous radiofrequency and it should be used only in selected cases [187]. Similar conclusions have been also drawn for cervical radicular pain and lumbar face pain [187]. On the opposite, intradiscal PRF seems to be a promising technique in case of discogenic pain, radiculitis, or spinal stenosis, although further studies are still required to confirm this indication [187].

#### Percutaneous electrical nerve stimulation

Percutaneous Electrical Nerve Stimulation (PENS) is a procedure using thin needles inserted through the skin to deliver electrical impulses to specific nerves or tissues [188]. The objective is to alleviate persistent and chronic pain by stimulation of peripheral sensory nerves through the insertion of needles in the outer layers of the skin, characterized by high resistance [188].

Today, the evidence in favor of PENS is quite weak. PENS (alone or in adjunct to other treatments) was shown to not provide a sufficient advantage in the control of musculoskeletal pain [189]. In one study including 17 patients with neuropathic pain due to spinal cord injury, PENS reduced the symptoms in 63% of cases [190]. However, no other studies have been conducted so far in this population, and the indication still lacks clear evidence. Another trial conducted in a population of patients with chronic low back pain secondary to degenerative disk disease, PENS was more effective in reducing pain and post-treatment function, as compared to Transcutaneous Electrical Nerve Stimulation (TENS) or other exercise therapies [191]. Finally, in a randomized double-blind crossover trial on 31 patients with neuropathic chronic pain, preliminary findings indicated that PENS may offer effective short-term pain relief [192].

Unfortunately, the underlying mechanisms of action of PENS are still few investigated, although they are thought to rely in the Gate Control Theory [7]. Despite its potential benefits in pain management, the precise indications for PENS, as well as the most effective treatment parameters, remain topics of discussion and investigation within the medical community. Additionally, comparative studies evaluating PENS against other therapeutic modalities are limited, leaving uncertainties about its relative efficacy and place in treatment algorithms [189, 193, 194]. To address these gaps in knowledge, future research efforts should prioritize well-designed studies that investigate the clinical utility of PENS across various pain conditions. These studies should aim to delineate the specific patient populations most likely to benefit from PENS, identify optimal stimulation parameters, and compare its efficacy and safety to other established treatments [189, 193, 194]. Furthermore, incorporating mechanistic studies to elucidate the underlying pathways through which PENS exerts its therapeutic effects can provide valuable insights into its mode of action and potential advantages over alternative interventions.

#### Transcranial direct current stimulation

Transcranial Direct Current Stimulation (tDCS) involves the non-invasive delivery of low-intensity electrical currents to specific brain regions for a duration of 20–30 min. These currents induce polarity-dependent shifts in resting membrane potential, thereby modulating neuronal activity at the site of stimulation and its associated structures [195, 196]. Typically, two electrodes, one anode positioned over the primary motor cortex and the other a cathode over the contralateral supraorbital region, are placed on the scalp. This setting generates a current flow through the prefrontal cortex, the cingulate cortex, the insula, and deeper structures such as thalamic and brainstem nuclei. The current flow modulates the rest membrane potential of axons, promoting the alleviation of pain [197].

To date, the efficacy of tDCS is limited to treatment of chronic neuropathic pain secondary to spinal cord injury [195] or for peripheral neuropathic pain [198]. Another recent meta-analysis has demonstrated that tDCS does not reduce the pain and related disability in non-specific chronic low-back pain syndrome, limiting the support for its use in this field [199]. Furthermore, a recent systematic review has assessed the efficacy of tDCS in migraine, reporting a positive effect on treatment success, although the included studies were quite heterogeneous and the sample size was small; therefore, these limitations precluded any definitive conclusion on the real efficacy of tDCS in this field of application [200].

#### Transcranial magnetic stimulation

Repetitive Transcranial Magnetic Stimulation (rTMS) is a non-invasive neurostimulation technique, applying a magnetic field externally to the scalp to induce electrical currents in targeted regions of the brain beneath the coil [201]. TMS operates on Faraday's principle of electromagnetic induction, where a rapid electric current passing through a coil for a brief duration, typically 1 ms, generates a potent electromagnetic field [202]. In TMS, the magnetic field is painlessly administered to the scalp, and cortical axons serve as the electrical conductors that receive the induced electric current [202].

The analgesic effect is obtained by stimulating the primary motor cortex (precentral gyrus), with a stimulation frequency ranging between 5 to 20 Hz, contralaterally to the side of pain localization [203, 204]. Therefore, rTMS has the capacity to regulate cortical hyperexcitability and pain pathways, including descending inhibitory pathways [205, 206]. These pathways exert an influence on supraspinal pain tracts and regions of the brain associated with social-affective functions, such as the right temporal lobe [207, 208].

rTMS has been applied in different populations of patients with chronic pain. In patients with migraine, rTMS decreases the intensity of the attack soon after the treatment application, particularly in those patients with cephalgia after traumatic brain injury [209]. High frequency rTMS on motor cortex has also proved to provide a positive temporary effect on pain in patients with neuropathic chronic pain correlated to phantom limb [210] and to manage pain alongside cognition and sleep disturbances in patients of fibromyalgia [211].

Finally, rTMS provides the additional purpose of anticipating the individual responsiveness of patients who may undergo subsequent implantation of a device for MCS [212]. In fact, a preoperative trial utilizing rTMS has demonstrated the ability to predict satisfactory pain relief in approximately 80 to 90% of cases where MCS implantation is performed [213, 214].

#### Transcutaneous electrical nerve stimulation

Transcutaneous Electrical Nerve Stimulation (TENS) is a non-invasive therapeutic technique based on the application of low-voltage electrical currents to the skin surface, through electrodes placed on or near the area of pain or along the nerve pathways [215]. The principles of TENS stay in the Gate Control Theory (see above) [7].

TENS is commonly employed for various types of pain. TENS has been studied to treat chronic low back pain. Two large randomized controlled trials were firstly conducted with contrasting results, mainly due to the different design, population, and modulation settings [216, 217]. A very recent systematic review, including the whole literature on this topic (i.e. 17 randomized controlled trials with a total of 1027 adults), has suggested that TENS may marginally reduce the perceived chronic low back pain for a short period, to an extent that cannot be considered clinically relevant [218]. TENS has also been tested in 43 patients with fibromyalgia, demonstrating that one 30-min application improved the pain perception during movement, but not at rest [219]. Then, several studies have been conducted so far in patients with fibromyalgia; the most recent pooled data analysis suggests that TENS improves pain symptoms in this population of patients [220]. A modified system for TENS specifically developed to stimulate the terminal supraorbital branch of the trigeminal nerve has been also demonstrated to prevent [221] or to treat attacks of episodic migraine [222]. However, the current evidence is still weak to recommend its use in this scenario.

#### Transcranial focused ultrasound

Transcranial Focused Ultrasound (tFUS) for chronic pain represents an emerging non-invasive neurostimulation method that utilizes focused ultrasound waves to modulate brain activity for therapeutic purposes [223]. This technique is characterized by a high spatial resolution, targeting any site of the peripheral or central nervous system [223].

tFUS induces mechanical vibrations and thermal effects influencing the neurons excitability, altering the synaptic transmission, and affecting the excitatory/inhibitory balance of neurotransmitters [224]. tFUS may also release endorphins or other neurochemicals involved in pain modulation [224]. In the application for chronic pain treatment, tFUS is centered to specific areas involved in the pain perception, such as the somatosensory cortex, thalamus, or limbic system [225–227].

Today, tFUS is approved in Europe for neuromodulation in neuropathic chronic pain not responsive to conventional pharmacological treatments [228]. Indeed, data available are limited to chronic neuropathic pain, phantom limb pain, neuropathic pain related to spinal cord injury in clinical and preclinical settings [229–231].

### Future perspectives of neurostimulation

Although the growing literature investigating the complexity of pain transmission and the biochemical effects of neurostimulation, clear indications and strong recommendations still lack. The complexity of anatomical and physiological pathways of pain modulation, together with the various etiopathogenesis and pathophysiology of different forms of chronic pain, should probably require a personalized approach to neurostimulation.

Patients can develop chronic pain through various pathophysiological pathways. However, any single treatment, whether pharmacological or involving neurostimulation, has a finite number of mechanisms of action. Consequently, it may only be effective for a specific subset of patients. Monotherapy, whether it's pharmacological, neurostimulatory, surgical, or psychological, is thus likely to result in some individuals who do not respond adequately. These individuals, termed "non-responders," have underlying disease mechanisms that do not align with the mechanism of action of the chosen therapy [212, 232].

Novel approaches to neurostimulation, inspired by the principles of the personalized and precision medicine, have been proposed for other diseases, like tinnitus [233–237], Parkinson’s disease [238, 239], epilepsy [240] and depression [241, 242]. A recent publication explores the reasoning and basis for personalized electrical neurostimulation in individuals suffering from chronic neuropathic pain [212].Noninvasive methods of neurostimulation may predict the response of every single patient to more invasive techniques such a MCS or DBS [212]. Beside the prediction of responsiveness to a specific treatment, the study of clinical phenotypes [243], brain networks and oscillatory patterns [244–247], and patient’s genotype with the corresponding single nucleotide polymorphism [248–251] may also guide the clinician in the adoption of the best strategy for every single patient. In addition, the differences in neuropathophysiological processes underlying various painful syndromes should be taken into consideration when selecting a neurostimulation strategy, as recently suggested [252].

It should also be considered that technical advances (such as microelectronics, feedback-based system design, biomimetic stimulation patterns), and the integration of genomics with device-based therapies are revolutionizing the conceptualization of neuromodulation, with a trend towards miniaturization and noninvasive therapies [253].

In this complex scenario, we believe that the scientific literature should be directed on the way to better clarify those underlying anatomical and pathophysiological mechanisms that are still obscure. Once these bases are clearer, more detailed, and better focused studies could be specifically designed to assess and to define the criteria of choice of one technique over another one, for every single chronic pain manifestation.

Meanwhile, the approach to a patient suffering from chronic pain necessitating neurostimulation demands meticulous evaluation, taking into account the underlying condition, individual characteristics (including psychological factors), and prior treatment modalities. Given the absence of comprehensive cost-effectiveness studies, except for SCS, physicians may opt to transition from non-invasive to more invasive techniques known to be efficacious for that particular painful condition.

## Conclusions

The electrical neuromodulation of pain comprises various techniques targeting distinct neural pathways involved in pain modulation. Despite numerous studies and trials, determining the most effective technique for each patient remains challenging. Patient selection plays a pivotal role in achieving optimal outcomes, considering factors such as the specific type and origin of chronic pain. Additionally, the invasiveness of the chosen treatment should align and be proportional with the patient's clinical status. Further trials are essential to refine these considerations. Meanwhile, a personalized approach to neurostimulation should guide clinicians in selecting the most appropriate treatment for individual patients.

## Data Availability

No datasets were generated or analysed during the current study.

## Abbreviations

5-HT: 5-Hydroxytryptamine
AMPA: Amino-3-hydroxy-5-methyl-4-isoxazole-propionic acid
CGRP: Calcitonin Gene-Related Peptide
CNS: Central Nervous System
DBS: Deep Brain Stimulation
DRG: Dorsal Root Ganglion
GABA: Gamma-Aminobutyric Acid
IASP: International Association for the Study of Pain
MCS: Motor Cortex Stimulation
NMDA: N-Methyl-D-Aspartate
PAG: PeriAqueductal Grey
PENS: Percutaneous Electrical Nerve Stimulation
PNS: Peripheral Nerve Stimulation
PRF: Pulsed Radiofrequency Therapy
rTMS: Repetitive Transcranial Magnetic Stimulation
RVM: RostroVentral Medulla
SCS: Spinal Cord Stimulation
tDCS: Transcranial Direct Current Stimulation
TENS: Transcutaneous Electrical Nerve Stimulation
tFUS: Transcranial Focused Ultrasound
VNS: Vagus Nerve Stimulation
